# Neuroendocrine Differentiation in Conventional Colorectal Adenocarcinomas: Incidental Finding or Prognostic Biomarker?

**DOI:** 10.3390/cancers13205111

**Published:** 2021-10-12

**Authors:** Björn Konukiewitz, Atsuko Kasajima, Maxime Schmitt, Kristina Schwamborn, Tanja Groll, Felix Schicktanz, Claire Delbridge, Lisa Marie Schütze, Dirk Wilhelm, Corinna Lang, Sebastian Lange, Sebastian Foersch, Paul Jank, Katja Steiger, Alexander von Werder, Carsten Denkert, Wilko Weichert, Günter Klöppel, Moritz Jesinghaus

**Affiliations:** 1 Department of Pathology, Universitätsklinikum Schleswig-Holstein, Campus Kiel, Christian-Albrechts-Universität zu Kiel, 24105 Kiel, Germany; Bjoern.Konukiewitz@uksh.de; 2 Department of Pathology, Technical University of Munich, 81675 München, Germany; atsuko.kasajima@tum.de (A.K.); maxime-schmitt@gmx.net (M.S.); kschwamborn@tum.de (K.S.); tanja.groll@tum.de (T.G.); felix.schicktanz@tum.de (F.S.); c.delbridge@tum.de (C.D.); glohlisa@aol.com (L.M.S.); corinna.maria.lang@icloud.com (C.L.); katja.steiger@tum.de (K.S.); wilko.weichert@tum.de (W.W.); guenter.kloeppel@tum.de (G.K.); 3 Department of Surgery, Klinikum Rechts der Isar, Technical University of Munich, 81675 München, Germany; dirk.wilhelm@tum.de; 4 II Medizinische Klinik, Klinikum Rechts der Isar, Technical University of Munich, 81675 München, Germany; sebastian.lange@tum.de (S.L.); alexandervon.werder@tum.de (A.v.W.); 5 Department of Pathology, University Hospital Mainz, 55131 Mainz, Germany; sebastian.foersch@unimedizin-mainz.de; 6 Department of Pathology, University Hospital Marburg, 35403 Marburg, Germany; paul.jank@uni-marburg.de (P.J.); carsten.denkert@uni-marburg.de (C.D.)

**Keywords:** neuroendocrine differentiation, colorectal adenocarcinomas, MANEC

## Abstract

**Simple Summary:**

Colorectal MANECs are highly aggressive carcinomas defined by a distinct neuroendocrine morphology and positivity for synaptophysin in the neuroendocrine component. It is unclear whether a neuroendocrine differentiation in conventional adenocarcinomas without a suggestive morphology is of clinical relevance. We tested 1002 conventional colorectal carcinomas with a non-neuroendocrine morphology for synaptophysin expression and correlated the results with clinicopathological characteristics as well as patient survival and compared the survival characteristics of synaptophysin expression groups to those of true MANECs. We found no survival differences between synaptophysin expression groups within conventional colorectal adenocarcinomas. MANECs, on the other hand, showed significantly worse survival characteristics. Our data suggest that synaptophysin expression in conventional colorectal adenocarcinomas is of minor prognostic relevance and that conventional adenocarcinomas with a diffuse synaptophysin expression should not be classified as MANECs.

**Abstract:**

Background: Colorectal mixed adenoneuroendocrine carcinomas (MANECs) are clinically highly aggressive neoplasms. MANECs are composed of variable adenocarcinoma components combined with morphologically distinct neuroendocrine carcinoma components, which are confirmed by synaptophysin immunohistochemistry, the gold standard marker of a neuroendocrine differentiation. However, the biological behavior of adenocarcinomas that express synaptophysin but do not show a typical neuroendocrine morphology remains unclear. Methods: We investigated synaptophysin expression in 1002 conventional colorectal adenocarcinomas and correlated the results with clinicopathological characteristics and patient survival and compared the survival characteristics of synaptophysin expression groups to MANECs. Results: Synaptophysin expression in conventional colorectal adenocarcinomas was associated with a shortened disease-free survival (*p* = 0.037), but not with overall survival or disease-specific survival (DSS) in univariate analyses and without any survival impact in multivariate analyses. Patients with “true” MANECs, on the other hand, showed a significantly shorter survival than all conventional adenocarcinomas with or without synaptophysin expression in uni- and multivariate analyses (e.g., multivariate DSS: *p* < 0.001, HR: 5.20). Conclusions: Our study demonstrates that synaptophysin expression in conventional colorectal adenocarcinomas, in contrast to MANECs, is not associated with a significantly poorer clinical outcome when compared to adenocarcinomas without synaptophysin expression. Furthermore, our data suggest that conventional adenocarcinomas with a diffuse synaptophysin expression should not be classified as MANECs, also strongly arguing that synaptophysin testing should be reserved for carcinomas with an H&E morphology suggestive of a neuroendocrine differentiation.

## 1. Introduction

Epithelial tumors composed of a neuroendocrine and a non-neuroendocrine component are called mixed neuroendocrine–non-neuroendocrine neoplasms (MiNENs) [1]. In the colon, they usually present as mixed adenoneuroendocrine carcinomas (MANEC), in which the adenocarcinoma component is combined with either a large cell or, rarely, a small cell neuroendocrine carcinoma (NEC). Genetically, colorectal MANECs as well as NECs have been found to be closely related to conventional colorectal adenocarcinomas, as they share crucial driver mutations [2,3]. However, MANECs differ from conventional colorectal carcinomas in their prognosis. In a recent study of a large cohort of more than 1000 colorectal carcinomas, we found that MANEC patients have a significantly worse clinical outcome than all patients with the other colorectal adenocarcinoma subtypes that are listed in the 2019 WHO Classification of Tumors of the Digestive System (WHO) [4]. They behave more like NECs and are therefore often treated like NECs [5,6]. Therefore, MANECs have to be correctly identified to be reliably distinguished from conventional colorectal adenocarcinomas. This is usually easy, since the histological component suggestive of a neuroendocrine differentiation is recognizable in many cases on H&E-stained sections [1]. However, it can be difficult in conventional colorectal adenocarcinomas that only reveal their neuroendocrine differentiation when immunohistochemically stained for synaptophysin, the immunohistochemical gold standard for the detection of neuroendocrine differentiation [7,8], which represents an integral membrane glycoprotein that is found in presynaptic vesicles of neurons as well as normal neuroendocrine epithelial cells (e.g., pancreatic islets) [9]. The neuroendocrine cells are usually found scattered in the mucin producing epithelium of the conventional adenocarcinomas and their numbers do not exceed the 30% threshold level that arbitrarily separates colorectal MANEC from colorectal adenocarcinoma with a neuroendocrine component [10]. However, there are occasional cases in which the number of synaptophysin-expressing cells in the epithelium of the neoplastic glands is so high that it is close to or even exceeds the 30% threshold level. Since such observations raise the question of the prognostic and clinical significance of neuroendocrine differentiation in these otherwise histologically inconspicuous conventional adenocarcinomas, a number of studies have dealt with this problem, but so far produced controversial results. While the extent of neuroendocrine differentiation was prognostically relevant in some studies, other studies were unable to confirm this statement [11,12,13,14,15,16,17,18,19]. Therefore, the prognostic assessment of colorectal adenocarcinomas with a neuroendocrine differentiation that is only demonstrable by immunohistochemistry remains a problem for diagnostic pathologists, especially if the neuroendocrine cell number appears to exceed the 30% cut-off level.

In this study, we investigated the frequency and extent of neuroendocrine differentiation, identified by synaptophysin expression, in a large cohort of 1013 colorectal carcinomas (1002 “conventional” adenocarcinomas with a non-neuroendocrine morphology on H&E sections and 11 colorectal MANECs) and correlated it with clinicopathological features and survival. Specifically, the following questions were addressed: (1) What is the frequency and extent of a neuroendocrine differentiation demonstrated by synaptophysin immunohistochemistry in conventional colorectal adenocarcinomas with a non-neuroendocrine morphology? (2) Are these colorectal carcinomas associated with certain clinicopathological parameters? (3) Are there significant differences in patient survival compared to, on the one hand, conventional adenocarcinomas without an immunohistochemically detectable neuroendocrine differentiation, and on the other hand, to typical colorectal MANECs?

## 2. Materials and Methods

### 2.1. Study Population

A total of 1002 colorectal adenocarcinomas and 11 colorectal MANECs from patients who underwent surgical resection between 1997 and 2019 at the University Hospital rechts der Isar of the Technical University of Munich were analyzed. All patients with colorectal carcinomas from this time span with fully available clinicopathological/survival data and with available tumor tissue on the Tissue Micro Array were included in this study. Formalin-fixed paraffin-embedded (FFPE) tumor samples from the tumor center and the invasive margin were assembled into the used tissue microarray (TMA) using a fully automated Tissue Microarrayer (TMA Grandmaster, sysmex, Budapest, Hungary) with a core size of 2 mm. All samples of a respective tumor region were extracted from areas harboring a high burden of invasive carcinoma, which were marked by an experienced pathologist (M.J.). Other tumors of the colorectal system (e.g., neuroendocrine tumors, non-epithelial tumors, etc.) were excluded. One case of an undifferentiated carcinoma from the original cohort was also excluded to avoid statistical bias. The clinicopathological characteristics as well as survival data for all patients were extracted from the Munich Cancer Registry and from hospital records. For overall survival (OS), all recorded patient deaths were noted. For disease-specific survival (DSS), only tumor-associated deaths were recorded as events. For disease-free survival (DFS), loco-regional or distant recurrence was noted as an event. Endpoints of all survival comparisons were either events or a loss of follow-up before 120 months, in which case the patients were censored at the time of the last available entry regarding the specific patient. All patients alive after 120 months were also censored. OS/DSS/DFS times were calculated using the date of the primary surgery as a starting point. The treatment concepts of included patients followed internal policies, which were based on the given German guidelines at the time of diagnosis, generally meaning that all patients were intended to receive stage-adapted treatment. Most of these tumors (1997–2018) were also examined in a recent study on incidence and critical relevance of morphological parameters in colorectal carcinoma subtypes as defined by the 2019 WHO classification of tumors of the digestive system [4].

The microsatellite status (MSI) was determined in the previous study [4], where all carcinomas were classified and subtyped according to the criteria of the 2019 WHO classification of tumors the digestive system, and pathological staging was reassessed using the current TNM classification of malignant tumors [1,20]. The detailed characteristics of the cohort, including age, sex, TNM, UICC-stage, resection-status, MSI-status, WHO grade, localization and tumor type, are depicted in Supplementary Table S1. This study was approved by the local ethics committee of the Technical University of Munich (reference number: 252/16 s).

#### 2.1.1. Histomorphological Characterization

**Figure 1:**
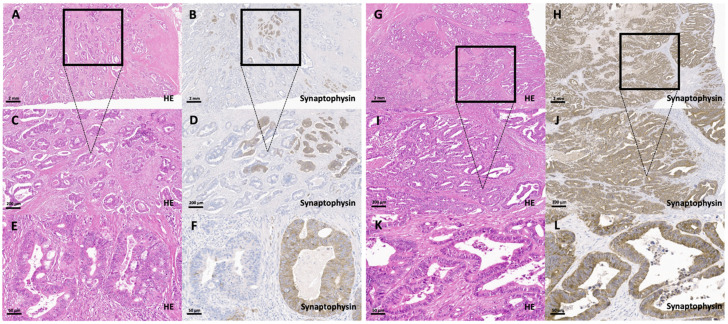
Synaptophysin-expressing groups in conventional colorectal adenocarcinomas with a non-neuroendocrine morphology. (**A**–**F**) Conventional colorectal adenocarcinoma with a non-neuroendocrine morphology (partial synaptophysin expression group; 10–29%) on H&E (**A** (2×), **C** (20×), **E** (40×)) and synaptophysin staining (**B** (2×), **D** (20×), **F** (40×)) with a group of synaptophysin-positive cells accounting for 15% of the whole tumor. (**E**–**H**) Conventional colorectal adenocarcinoma with a non-neuroendocrine morphology with a diffuse synaptophysin expression in all tumor cells on H&E (**G** (2×), **I** (20×), **K** (40×)) and synaptophysin staining (**H** (2×), **J** (20×), **L** (40×)).

**Figure 2:**
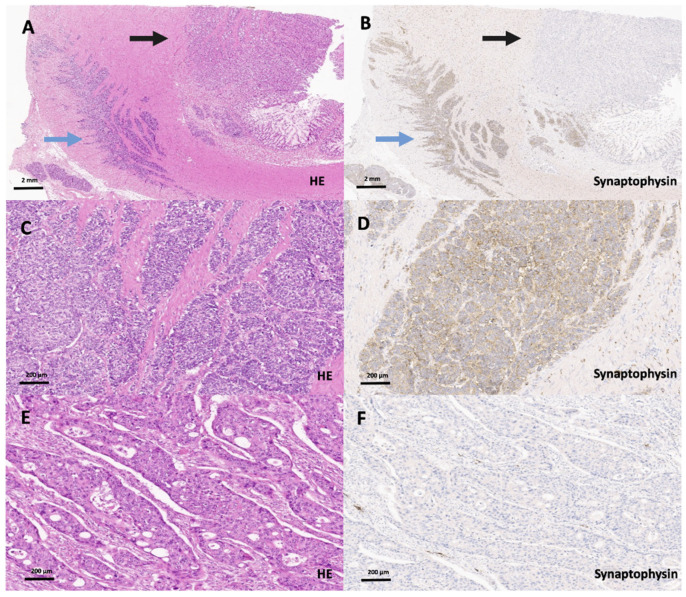
Scanning magnification (**A**, HE, 2×; **B**, synaptophysin, 2×) of a true colorectal MANEC (blue arrow: NEC, black arrow: adenocarcinoma component). Higher magnification of the NEC component on H&E (**C**, 20×) and synaptophysin staining (**D**, 20×) showing the typical NEC morphology. Higher magnification of the poorly differentiated, synaptophysin-negative adenocarcinoma component (**E**, HE, 20×; **F**, synaptophysin, 20×) of this colorectal MANEC that does not show a neuroendocrine histomorphology.

Full block H&E slides from 1013 colorectal carcinomas that were (mostly) part of a previously published collective were rescreened on full block slides at the beginning of this study [4], where the carcinomas were re-classified in accordance with the subtypes listed in the 2019 WHO classification of tumors of the digestive system. Tumors that were not part of the previous cohort but added to the collective were classified as described previously [4]. The final investigated cohort comprised 1002 colorectal adenocarcinomas of various subtypes that showed no morphologic features suggestive of a neuroendocrine carcinoma (Figure 1). Eleven colorectal cancers were diagnosed as MANECs on full block slides as they showed adenocarcinomas that were mixed with a tumor component >30% that was morphologically suggestive of a neuroendocrine carcinoma and that expressed synaptophysin (and Chromogranin A), according to current WHO guidelines (Figure 2). These 11 colorectal MANECs were used as a statistical control group for further analyses.

#### 2.1.2. Immunohistochemistry

The TMA was stained with synaptophysin (polyclonal, Ventana medical systems, Tucson, AZ, USA, prediluted). A cytoplasmic synaptophysin expression was considered specific. A scattered expression pattern was defined by us as a discontinuous expression of synaptophysin while a staining of more than 50 continuous cells was stated as block-like (Supplementary Figure S1). Finally, for each core, the synaptophysin-positive cells were counted and the mean percentage of positive cells for both cores was assigned for each tumor.

### 2.2. Statistics

Statistical analyses were performed using SPSS version 26 (SPSS Institute, Chicago, IL, USA). Associations were calculated with an χ2 test as well as an χ2 test for trends and Fisher’s exact test. The Bonferroni method was used to correct for multiple testing. Survival probabilities were plotted with the Kaplan–Meier method, and a log-rank test was used to probe for the significance of differences in survival. Multivariate survival analyses (including age, gender, UICC stage, synaptophysin expression groups in conventional adenocarcinomas (+typical MANECs), WHO grade) were performed with the Cox proportional hazard model. *p*-values ≤ 0.05 were considered significant. All statistical tests were performed two-sided.

## 3. Results

### 3.1. Clinicopathological Features and Survival

The cohort of 1013 colorectal carcinomas included 1002 colorectal adenocarcinomas without histological features suggestive of neuroendocrine differentiation (“conventional colorectal adenocarcinomas”). Eleven carcinomas had a neuroendocrine carcinoma component that showed a typical neuroendocrine histology (MANECs), characterized by a solid and diffuse growth pattern with necrotic foci and cells containing large vesicular nuclei with nucleoli and easy recognizable mitoses. The median age of patients at diagnosis was 69 years (range: 9–87). There were 581 male patients and 432 female patients. As expected, pTNM/UICC stage, WHO grade and resection status were significantly associated with OS, DSS and DFS (Supplementary Table S1) [4].

### 3.2. Synaptophysin Expression in Conventional Colorectal Adenocarcinomas without Histological Features Suggestive of a Neuroendocrine Differentiation

The 1002 conventional adenocarcinomas included 763 synaptophysin-negative (76%) and 239 synaptophysin-positive tumors. The expression of synaptophysin (Table 1) ranged from single scattered synaptophysin-positive tumor cells to a strong and diffuse expression in almost all tumor cells. The tumors of the first and largest group (126/239; 53%) had only single positive cells which accounted for less than 1% of the tumor cell population. The second largest group contained adenocarcinomas with a range from >1–9% of scattered synaptophysin-positive tumor cells (72/239; 30%). The third group encompassed 15 carcinomas with synaptophysin-positive cells ranging from 10–29% (15/239; 6%) and the fourth group had 14 carcinomas with a diffuse synaptophysin staining of 30–99% of all tumor cells (14/239; 6%). The fifth and last group summarized 12/239 (5%) adenocarcinomas with diffuse and intense synaptophysin staining of almost all cells of the neoplastic glands. After initial statistical analysis (and under consideration of the used WHO threshold) [10], four subgroups of adenocarcinomas were compiled: synaptophysin-negative adenocarcinomas (763/1002), adenocarcinomas with scattered (<1–9% of the tumor cells; 198/1002), partial (synaptophysin expression in 10–29% of the tumor cells; 15/1002, Figure 1) and diffuse (synaptophysin expression in 30–100% of the tumor cells; 26/1002, Figure 1) synaptophysin expression.

### 3.3. Correlation of Synaptophysin Expression in Conventional Colorectal Adenocarcinomas with Clinicopathological Data

Scattered synaptophysin-expressing cells were seen in all subtypes. The diffuse expression pattern was weakly associated with adenocarcinoma subtypes (*p* = 0.05). Synaptophysin positivity was significantly associated with lymph node metastases (*p* < 0.001), UICC stage (*p* = 0.01) and lymphatic invasion (*p* = 0.02) but not with age, sex, T stage, vascular invasion, perineural invasion, resection margin, microsatellite status, localization or WHO grade (Table 1).

### 3.4. Correlation of Synaptophysin Expression in Conventional Colorectal Adenocarcinomas with Survival Parameters

**Figure 3:**
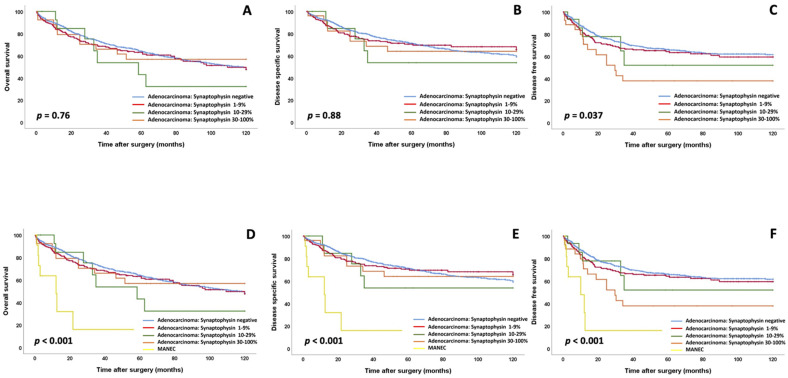
(**A**–**C**) Overall survival, disease-specific survival and disease-free survival for synaptophysin expression groups in conventional adenocarcinomas with a non-neuroendocrine morphology (**A**–**C**); note that there are no survival differences between synaptophysin expression groups in conventional adenocarcinomas and only a slightly reduced disease-free survival for conventional adenocarcinomas with a synaptophysin expression of 30% or more. (**D**–**F**): Overall survival, disease-specific survival and disease-free survival for synaptophysin expression groups in conventional adenocarcinomas compared to typical MANECs (**D**–**F**); please note that while the conventional adenocarcinomas show similar survival characteristics, the MANECs show significantly unfavorable survival characteristics.

In univariate analysis of the 1002 conventional colorectal adenocarcinomas, synaptophysin-expressing groups showed an association with DFS (*p* = 0.037), but not with OS or DSS (Figure 3, Supplementary Table S2). There was no impact on all survival parameters in specific stage groups (UICC stage I and II versus UICC stage III and IV) or clinicopathological subcohorts (low vs. high WHO grade, T stage groups, N stage groups, data not shown). In multivariate analysis (including age, sex, UICC stage and WHO grade), only UICC stage and WHO grade, but not synaptophysin expression, impacted OS, DSS and DFS (DFS: *p* = 0.49, Table 2; DSS: *p* = 0.23, OS: *p* = 0.16).

### 3.5. Survival of True Colorectal MANECs Compared to Colorectal Adenocarcinomas without Histological Features Suggestive of a Neuroendocrine Differentiation

The true MANECs showed a significantly worse clinical outcome (OS: *p* < 0.001; DSS: *p* < 0.001; DFS: *p* < 0.001; Table 3, Supplementary Table S3) compared to the group of diffuse synaptophysin-expressing conventional adenocarcinomas (e.g., mean DFS: 16.98 months vs. 77.81 months) in univariate analyses. These results were confirmed for all parameters by multivariate analyses including WHO grade, UICC stage, age and sex (e.g., DFS: *p* = 0.001, HR: 3.87, Table 3; DSS: *p* < 0.001, HR: 5.20, Supplementary Table S4; OS: *p* < 0.001, HR: 4.16).

## 4. Discussion

In a previous study [4], we demonstrated that colorectal MANECs with a histologically recognizable neuroendocrine carcinoma component confirmed by synaptophysin positivity are associated with a significantly poorer prognosis when compared to conventional adenocarcinomas NOS and other adenocarcinoma subtypes [4]. However, this study did not examine the clinical relevance of the expression of synaptophysin, the gold standard marker for the immunohistochemical confirmation of a neuroendocrine differentiation [21], in conventional colorectal adenocarcinomas without a histological pattern suggestive of neuroendocrine differentiation. In the current study, we screened 1002 conventional adenocarcinomas for synaptophysin expression and found that approximately a quarter of these tumors harbored synaptophysin-positive cells, albeit mostly as scattered tumor cells embedded in the epithelium of the neoplastic glands. A significant synaptophysin expression in at least 10% of the tumor cell population was only found in 4% of all cases, with more than half of them with an expression of at least 30% of the tumor cells, thereby reaching the immunohistochemical WHO threshold level qualifying a colorectal carcinoma for a MANEC [10].

The most important result of this study was that none of the synaptophysin-expressing groups of conventional colorectal adenocarcinomas (adenocarcinoma NOS and specific WHO subtypes) showed significantly different overall survival or disease-specific survival parameters compared to non-synaptophysin-expressing conventional colorectal carcinomas. In conventional adenocarcinomas with a synaptophysin expression of more than 30% of the tumor cell population, a slightly poorer disease-free survival was noted in univariate analysis, but this result was not confirmed by multivariate analysis including UICC stage, WHO grade, age and gender. Our data thus suggest that synaptophysin expression in conventional colorectal adenocarcinomas without any component suggestive of a neuroendocrine differentiation in H&E-stained sections is of minor prognostic relevance, at best.

In the next step, we compared the survival data of synaptophysin-expressing conventional adenocarcinomas with those of true colorectal MANECs. In uni- and multivariate analyses (including age, sex, UICC stage, WHO grade), we observed that the MANECs had a significantly shorter overall survival, disease-specific survival and disease-free survival than all synaptophysin-expressing adenocarcinomas, including conventional adenocarcinomas with diffuse synaptophysin expression in more than 30% of the cells of the neoplastic glands. These data suggest that the clinical relevance of synaptophysin expression in colorectal adenocarcinomas is strongly related to a histologically recognizable neuroendocrine component, usually with the features of a large cell neuroendocrine carcinoma. The composition of the exocrine and the neuroendocrine component to each other may differ from case to case but can morphologically be traced back to a collision, combined or amphicrine type in most cases [2,3].

Many studies investigated the prognostic impact of neuroendocrine differentiation in gastrointestinal carcinomas [12,14,17,18,19,22,23,24], and all studies showed that the expression of neuroendocrine markers such as synaptophysin is linked to a poor prognosis when the tumor has a histological pattern suggestive of neuroendocrine differentiation in H&E-stained sections. However, conflicting results were produced by studies that defined a neuroendocrine differentiation solely by immunohistochemistry regardless of the carcinoma morphology, either reporting poor prognosis [13], association with distant metastasis [14] or not showing any prognostic impact at all [17,18].

The correct recognition of MANECs is not only important for the assessment of the clinical course, but also for the therapeutic strategy that derives from this assessment, as the presence of a poorly differentiated neuroendocrine component usually qualifies these patients for specific chemotherapy regimens (often a combination of platinum derivatives and topoisomerase inhibitors such as Cisplatin and Etoposid) [5,6,25]. Nevertheless, our study has some limitations: this is a retrospective analysis, and the results of this paper should be validated in a prospective fashion. Furthermore, based on our data, we are not able to make further conclusions about the molecular underpinnings and possible differences in treatment response of synaptophysin-expressing conventional colorectal adenocarcinomas without a morphologically recognizable neuroendocrine component. Further studies including clinical trials are needed to address this issue.

## 5. Conclusions

In conclusion, we demonstrated that synaptophysin expression in conventional colorectal adenocarcinomas, in contrast to colorectal MANECs, is not associated with a significantly poorer clinical outcome when compared to conventional adenocarcinomas without synaptophysin expression. Therefore, our data strongly suggest that synaptophysin testing should be restricted to carcinomas whose morphology on H&E-stained sections indicates a neuroendocrine differentiation. It also means that conventional adenocarcinomas, in which the cells of the neoplastic glands diffusely express synaptophysin and exceed in number the 30% threshold level, should not be classified as MANECs.

## Figures and Tables

**Table 1:** Distribution and statistical associations to clinicopathological characteristics of synaptophysin expression groups in conventional adenocarcinomas with a non-neuroendocrine morphology.

		Overall	Adenocarcinoma Synaptophysin-Negative Cells	Adenocarcinoma 1–9% Synaptophysin-Positive Cells	Adenocarcinoma 10–29% Synaptophysin-Positive Cells	Adenocarcinoma 30–100% Synaptophysin-Positive Cells	*p*-Value
		1002 (100%)	763 (76%)	198 (20%)	15 (1%)	26 (3%)	
Age							0.46
	below median	485 (48%)	367 (48%)	102 (51%)	5 (33%)	11 (42%)	
	above median	517 (52%)	396 (52%)	96 (49%)	10 (67%)	15 (58%)	
Sex							0.52
	male	575 (57%)	431 (56%)	118 (60%)	8 (53%)	18 (69%)	
	female	427 (43%)	332 (44%)	80 (40%)	7 (47%)	8 (31%)	
pT							0.49
	1	73 (7%)	51(7%)	21 (11%)	0	1 (7%)	
	2	180 (18%)	136 (18%)	36 (18%)	4 (27%)	4 (18%)	
	3	558 (56%)	429 (56%)	104 (52%)	7 (46%)	18 (69%)	
	4	191 (19%)	147 (19%)	37 (19%)	4 (47%)	3 (19%)	
pN							<0.001
	0	560 (56%)	426 (56%)	120 (61%)	5 (33%)	9 (35%)	
	1	284(28%)	225 (29%)	46 (23%)	7 (47%)	6 (23%)	
	2	158 (16%)	112 (15%)	32 (16%)	3 (20%)	11 (42%)	
pM							0.09
	0	859 (86%)	663 (87%)	168 (85%)	11 (73%)	17 (64%)	
	1	143 (14%)	100 (13%)	30 (15%)	4 (27%)	9 (36%)	
UICC Stage							0.01
	1	201 (20%)	144 (19%)	51 (26%)	2 (13%)	4 (15%)	
	2	342 (34%)	269 (35%)	66 (33%)	2(13%)	5 (19%)	
	3	310 (31%)	245 (32%)	50 (25%)	7 (47%)	8 (31%)	
	4	149 (15%)	105 (14%)	31 (16%)	4 (27%)	9 (35%)	
Lymphatic							0.02
Invasion	not present	498 (49%)	381 (50%)	106 (54%)	4 (27%)	7 (27%)	
	present	504 (51%)	382 (50%)	92 (46%)	11 (73%)	19 (73%)	
Vascular							0.19
Invasion	not present	867 (86.5%)	663 (87%)	169 (86%)	15 (100%)	20 (77%)	
	present	135 (13.5%)	100 (13%)	29 (14%)	0	6 (23%)	
Resection							0.19
Margin	R0	933 (93%)	709 (93%)	188 (85%)	13 (87%)	23 (88%)	
	R1	41 (4%)	34 (4%)	3 (2%)	2 (13%)	2 (8%)	
	R2	28 (3%)	20 (3%)	7 (3%)	0	1 (4%)	
Localization							0.51
	right colon	488 (49%)	368 (48%)	96 (48%)	10 (67%)	14 (54%)	
	left colon	514 (51%)	395 (52%)	102 (52%)	5 (33%)	12 (46%)	
WHO Tumor Type							0.01
	Adenocarcinoma NOS	629 (63%)	480 (61%)	125 (62%)	8 (53%)	16 (61%)	
	Mucinous adenocarcinoma	86 (8%)	71 (9%)	13 (6%)	1 (7%)	1 (4%)	
	Signet-ring cell carcinoma	9 (1%)	6 (1%)	1 (1%)	0	2 (8%)	
	Medullary carcinoma	31 (3%)	24 (4%)	6 (3%)	1 (7%)	0	
	Micropapillary adenocarcinoma	128 (13%)	102 (13%)	17 (9%)	3 (20%)	6 (23%)	
	Serrated adenocarcinoma	88 (9%)	60 (8%)	25 (13%)	2 (13%)	1 (4%)	
	Adenoma-like adenocarcinoma	31 (3%)	20 (4%)	11 (6%)	0	0	
Microsatellite							0.19
Status	microsatellite stable	846 (84%)	634 (83%)	175 (88%)	13 (87%)	24 (92%)	
	microsatellite instable	156 (16%)	129 (17%)	23 (12%)	2 (13%)	2 (8%)	
WHO Grade							0.19
	low-grade	687 (68%)	520 (68%)	144 (73%)	8 (53%)	15 (69%)	
	high-grade	315 (32%)	243 (32%)	54 (27%)	7 (47%)	11 (31%)	

**Table 2:** Multivariate analysis for disease-free survival including synaptophysin expression groups in conventional adenocarcinomas with a non-neuroendocrine morphology, UICC stage, WHO grade, age and sex.

		HR (DFS)	Lower CI (95%)	Upper CI (95%)	*p*-Value
Conventional Adenocarcinoma Synaptophysin Subgroups					0.49
	Conventional adenocarcinoma synaptophysin negative	1.0			
	Conventional adenocarcinoma 1–9% synaptophysin positive	1.2	0.92	1.60	
	Conventional adenocarcinoma 10–29% synaptophysin positive	0.8	0.34	1.97	
	Conventional adenocarcinoma 30–100% synaptophysin positive	1.1	0.64	1.94	
WHO grade					0.01
	Low grade	1.00			
	High grade	1.34	1.07	1.69	
UICC Stage	I	1.00			<0.001
	II	2.16	1.30	3.50	
	III	3.94	2.48	6.25	
	IV	11.87	7.40	19.05	
Age group					0.72
	Below median	1.00			
	Median and above	1.05	0.84	1.32	
Sex	male	1.0			0.48
	female	1.08	0.86	1.35	

**Table 3:** Multivariate analysis for disease-free survival including synaptophysin expression groups in conventional adenocarcinomas with a non-neuroendocrine morphology compared to MANECs and UICC stage, WHO grade, age and sex.

		HR (DFS)	Lower CI (95%)	Upper CI (95%)	*p*-Value
Conventional Adenocarcinoma Synaptophysin Subgroups					0.001
versus MANEC/NEC					
	Conventional Adenocarcinoma synaptophysin negative	1.0			
	Conventional Adenocarcinoma 1–9% synaptophysin positive	1.20	0.91	1.59	
	Conventional Adenocarcinoma 10–29% synaptophysin positive	0.83	0.34	2.01	
	Conventional Adenocarcinoma 30–100% synaptophysin positive	1.12	0.65	1.96	
	MANEC/NEC	3.87	1.79	8.37	
WHO grade					0.011
	Low grade	1.00			
	High grade	1.34	1.07	1.68	
UICC Stage	I	1.00			<0.001
	II	2.10	1.31	3.44	
	III	4.17	2.52	6.33	
	IV	12.16	7.39	19.02	
Age group					0.61
	Below median	1.00			
	Median and above	1.05	0.86	1.36	
Sex	male	1.0			0.37
	female	1.14	0.83	1.31	

## Data Availability

All data relevant for this study are given with the main paper including figures, tables and the supplemental files. The tissue investigated for this study is archived in the Institute of Pathology of the Technical University of Munich.

## Supplementary Materials

The following are available online at https://www.mdpi.com/article/10.3390/cancers13205111/s1. Supplementary Table S1: Clinicopathological characteristics of the overall cohort including conventional adenocarcinomas and MANECs with respect to survival parameters (overall, disease-specific, disease-free survival). Supplementary Table S2: Impact of synaptophysin expression groups on survival parameters in conventional adenocarcinomas with a non-neuroendocrine morphology (log-rank test). Supplementary Table S3: Impact of synaptophysin expression groups on survival parameters in adenocarcinomas with a non-neuroendocrine morphology compared to MANECs (log-rank test). Supplementary Table S4: Multivariate analysis for disease-specific survival including synaptophysin expression groups in conventional adenocarcinomas with a non-neuroendocrine morphology compared to MANECs including UICC stage, WHO grade, age and sex. Supplementary Figure S1: Overview of synaptophysin staining patterns.
