# Supplementary material for: Neuroendocrine Differentiation in Conventional Colorectal Adenocarcinomas: Incidental Finding or Prognostic Biomarker?
Source: Cancers (Basel). 2021 Oct 12;13(20):5111. doi: 10.3390/cancers13205111 (PMC8533893; doi:10.3390/cancers13205111)
Supplement: Supplementary file 1 [file cancers-13-05111-s001.zip › Supplementary/Supplementary Figure 1.pdf]

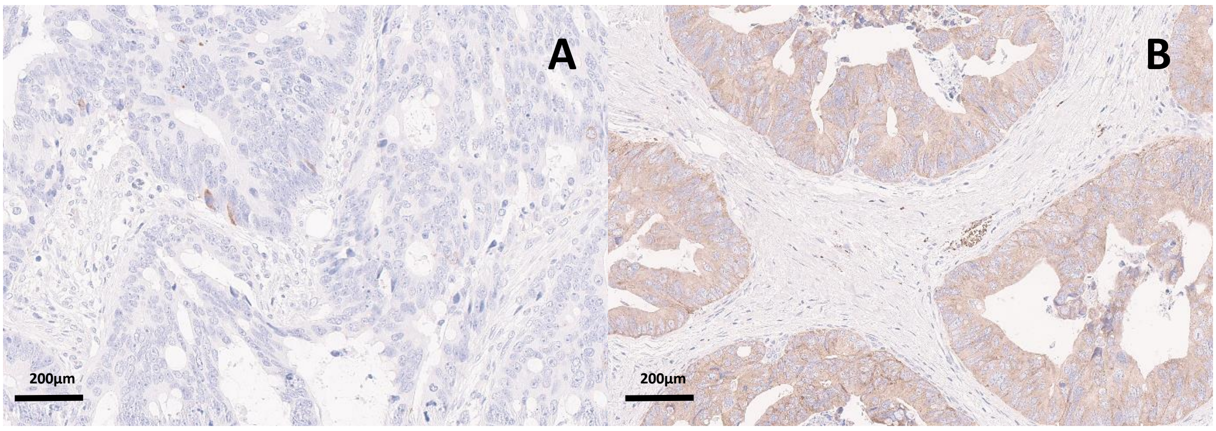

Supplementary Figure 1:

A: Conventional colorectal carcinoma with a scattered, discontinous expression of Synaptophysin (20x) in only a few cells.

B: Block like expression of Synaptophysin in a conventional colorectal carcinoma (20x) with more than 50 cells that are continuously positive.
